# Intralobular Distribution of Vitamin A-Storing Lipid Droplets in Hepatic Stellate Cells with Special Reference to Polar Bear and Arctic Fox

**DOI:** 10.1186/1476-5926-2-S1-S16

**Published:** 2004-01-14

**Authors:** Nobuyo Higashi, Katsuyuki Imai, Mitsuru Sato, Takeya Sato, Naosuke Kojima, Mitsutaka Miura, Heidi L Wold, Jan Øivind Moskaug, Trond Berg, Kaare R Norum, Norbert Roos, Kenjiro Wake, Rune Blomhoff, Haruki Senoo

**Affiliations:** 1 Department of Anatomy, Akita University School of Medicine, Akita 010-8543, Japan; 2 Liver Research Unit, Minophagen Pharmaceutical Co. Ltd., Tokyo 160-0004, Japan; 3 Institute for Nutrition Research, Faculty of Medicine, University of Oslo, Norway; 4 Division of Molecular Biology, Institute of Biology, University of Oslo, Norway; 5 Electronmicroscopical Unit for Biological Sciences, Faculty of Mathematics and Natural Sciences, University of Oslo, Norway

## Abstract

We examined the liver of adult polar bears, arctic foxes, and rats by gold chloride staining, fluorescence microscopy for the detection of autofluorescence of vitamin A, hematoxylin-eosin staining, staining with Masson's trichrome, Ishii and Ishii's silver impregnation, and transmission electron microscopical morphometry. The liver lobules of the arctic animals showed a zonal gradient in the storage of vitamin A. The density (i.e., cell number per area) of hepatic stellate cells was essentially the same among the zones. These results indicate that the hepatic stellate cells of the polar bears and arctic foxes possess heterogeneity of vitamin A-storing capacity in their liver lobules.

## Introduction

Hepatic stellate cells (vitamin A-storing cells, fat-storing cells, lipocytes, interstitial cells) are located in the perisinusoidal space of Disse and extend their thin fibrillar processes into this space [1,2]. Under physiological conditions, the hepatic stellate cells can store 80% of the total vitamin A in the whole body as retinyl esters in lipid droplets in the cytoplasm and play pivotal roles in regulation of vitamin A homeostasis [3]. We have reported that arctic animals such as polar bear store a large amount of vitamin A in hepatic stellate cells as compared to human or usual experimental animals such as rats or mice. However, the question as to the distribution of vitamin A-storing lipid droplets in hepatic stellate cells within the liver lobule remains unsettled. Therefore, we conducted the present study on polar bears and arctic foxes.

## Methods

**Figure 1:**
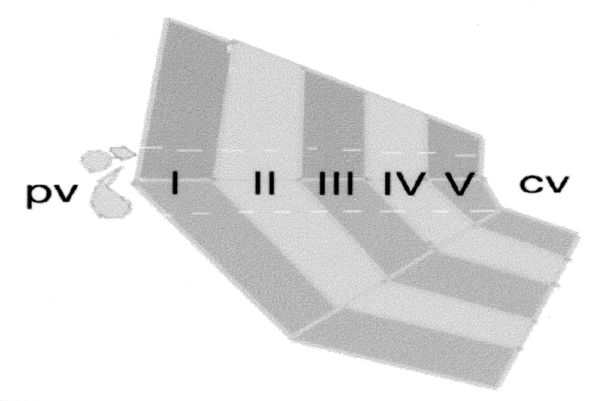
Zonal division of the liver lobule. To make a zonal morphometric analysis, we divided the liver lobule histologically into 5 zonal areas of equal widths (I, II, III, IV, and V) from the portal vein (pv) to the central vein (cv).

After having obtained permission to hunt animals from the District Governor of Svalbard, we caught 11 arctic foxes (*Alopex lagopus*) during the period from August 1996 to September 2001. Three polar bears (*Ursus maritimus*) were shot in self-defense in February and August 1998. We examined the liver of these animals by gold chloride staining, fluorescence microscopy for the detection of autofluorescence of vitamin A, hematoxylin-eosin staining, staining with Masson's trichrome, Ishii and Ishii's silver impregnation, and transmission electron microscopical morphometry using a division map of liver lobule for a zonal analysis (Figure 1). As a control, we examined the liver of rats in the same procedure.

## Results

**Figure 2:**
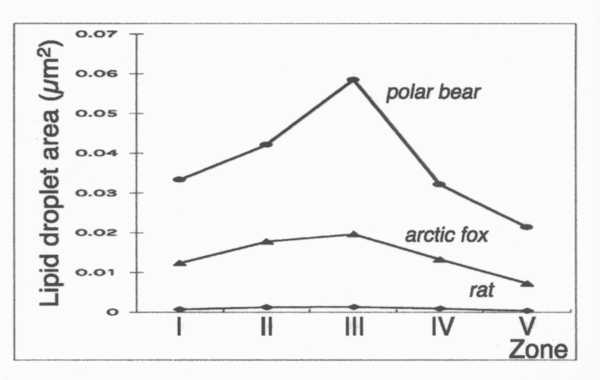
Intralobular zonal gradient of vitamin A-storage in hepatic stellate cells. Zonal gradient of vitamin A-storage is expressed as an asymmetric crescendo-decrescendo profile with a peak at zone III and a downward slope toward zone V. Graphs were plotted with the mean values, depicting the 3 animal species, namely (top to bottom), polar bear, arctic fox, and rat.

The liver lobules of the arctic animals showed a zonal gradient in the storage of vitamin A (Figure 2). The gradient was expressed as a symmetric crescendo-decrescendo profile starting at periportal zone, peaking at the middle zone, and sloping down toward the central zone in the liver lobule. The area of lipid droplets in the middle zone of polar bears was the largest in all zones evaluated. It was about 3 times as large as that of middle zone in arctic foxes, and about 43 times as large as that of the corresponding zone in rats. We also compared the cell density of stellate cells in each zone, but the cell density in each zone showed no significant differences (data not shown). The zonal differences revealed by light microscopical methods (data not shown) were consistent with electron microscopic morphometry. No pathological signs such as liver cirrhosis or hepatic fibrosis were observed in these animals (data not shown).

## Discussion

Results of this study indicate that arctic animals possess the intralobular heterogeneity of vitamin A-storage capacity in the hepatic stellate cells.

In earlier studies, a specific zonality for intralobular vitamin A-storage was reported. Although our results agree with other reports as to the presence of the heterogeneity of vitamin A-storing lipid droplets in the hepatic stellate cells in the liver lobule [4,5], the location of the highest-storage zone disagree with these reports. This discrepancy might be explained by the observation methods used in the earlier studies. Whereas those authors used only the light microscopical method [4,5], in the present study we examined all tissues by electron microscopy. Moreover, we certified that the tendency of vitamin A storage observed in liver lobules by light microscopy strongly supported that of intralobular heterogeneity revealed by electron microscopical morphometry.

The existence of the intralobular heterogeneity of vitamin A storage would relate to the number of hepatic stellate cells within the each zone or the maturation of hepatic stellate cells itself [6]. However, regarding the quantification of cell density in each zone, the average density calculated in all the animal species examined in our study did not differ among the zones. In previous papers we reported that hepatic stellate cells displayed a different response to extracellular matrix (ECM) components to change their shape and cellular functions [7]. In addition, the heterogeneity of ECM in the liver lobule [8] and the modulation of the cellular retinol-binding protein (CRBP) level by the extracellular collagen matrix in the hepatic stellate cells have been reported [9]. CRBP plays an important role in retinol metabolism and has also been reported to be indispensable for efficient retinyl ester synthesis and storage [10]. Furthermore, Kato et al. [11] has reported the existence of the intralobular heterogeneity of CRBP in the liver of rats.

Hence, we speculate that the intralobular distribution of CRBP in the liver of polar bears and arctic foxes may also show similar heterogeneity to that in the rats, and have effects on the intralobular heterogeneity of storing vitamin A in the livers.

## References

[B1] Wake K (1971). "Sternzelln" in the liver: Perisinusoidal cells with special reference to storage of vitamin A. Am J Anat.

[B2] Imai K, Sato T, Senoo H (2000). Adhesion between cells and extracellular matrix with special reference to hepatic stellate cell adhesion to three-dimensional collagen fibers. Cell Struct Funct.

[B3] Blomhoff R (1994). Vitamin A in Health and Disease. New York: Marcel Dekker.

[B4] Wake K, Sato T (1993). Intralobular heterogeneity of perisinusoidal stellate cells in porcine liver. Cell Tissue Res.

[B5] Zou Z, Ekataksin W, Wake K (1998). Zonal and regional differences identified from precision mapping of vitamin A-storing lipid droplets of the hepatic stellate cells in pig liver: A novel concept of addressing the intralobular area of heterogeneity. Hepatology.

[B6] Wake K, Motomatsu K, Ekataksin W (1991). Postnatal development of the perisinusoidal stellate cell in the rat liver. Proceeding of the Fifth International Symposium on Cells of the Hepatic Sinusoid: 1990 August 26Ø30.

[B7] Li Y, Sato M, Kojima N, Miura M, Senoo H (1999). Regulation role of extracellular matrix components in expression of matrix metalloproteases in cultured hepatic stellate cells. Cell Struct Funct.

[B8] Reid LM, Fiorino AS, Sigal SH, Bill S, Holst PA (1992). Extracellular matrix gradients in the space of Disse: relevance to liver biology. Hepatology.

[B9] Davis BH, Pratt BM, Madri JA (1987). Retinol and extracellular collagen matrices modulate hepatic Ito cell collagen phenotype and cellular retinol binding protein levels. J Biol Chem.

[B10] Ghyselinck NB, BØvik C, Sapin V, Mark M, Bonnier D, Hindelang C, Dierich A (1999). Cellular retinol-binding protein I is essential for vitamin A homeostasis. The EMBO Journal.

[B11] Kato M, Kato K, Goodman DS (1984). Immunocytochemical studies on the localization of plasma and of cellular retinol-binding proteins and of transthyretin (prealbumin) in rat liver and kidney. J Cell Biol.

